# Notes and News

**Published:** 1896-05-02

**Authors:** 


### NOTES AND NEWS.

(Collected and Communicated.)

### HOSPITAL MEETINGS.

The 36th Annual General Meeting of the Seaside Convalescent Hospital, Seaford, will be held on Wednesday, May 6th, at twelve noon, at 23, Gresham House, Old Broad Street, E.C.

Professor Roentgen has been created a Knight of one of the Bavarian Orders by the Prince Regent, and is now Professor von Roentgen. He has also been presented with the honorary degree of Doctor of Medicine by the University of Würsburg.

His Grace the Duke of Marlborough has accepted the presidency of the National Orthopædic Hospital, and, in company with the Duchess, will preside at the festival dinner at the Whitehall Rooms, Hotel Metropole, on Thursday, May 14th.

The Huxley Memorial now amounts to £2,296. Sufficient money has now been guaranteed to permit of the erection of a statue in the British Museum of Natural History for a medal at the Royal College of Science. A third object is now being appealed for, to help the furtherance of biological science. The exact scheme is not yet determined.

A cottage hospital was opened at Melksham last week. It is the gift of Mr. H. G. White, of Whitley, and is estimated to cost when entirely finished the sum of £1,000. A cottage hospital has also been erected at Wells through the munificence of the late Dean Plumptre, who left the sum of £2,000 to rebuild and refurnish the Cottage Hospital.

The fate of Gloucester has opened the vaccination question elsewhere, and many neglected enforcements of the Vaccination Act have been brought to light. At Eastbourne, a lady guardian had the courage to point out that all was not as it should be, and some annoyance was expressed that she should have opened the question just at the beginning of the season. But Eastbourne will have cause to be grateful to Miss Hall, if, by exposing the weak spot in time, she saves the watering-place from such a fate as Worthing, which is only now beginning to recover after the severe shock caused by another epidemic.

Liebig's Extract of Meat Company have given one hundred guineas towards the re-endowment fund of Guy's Hospital.

Measures are being taken to apply the Roentgen method of photography to military surgery, and it is expected that Africa will be the first field of operation in connection with the English army.

Two surgeons of the National Society for the Aid of the Sick and Wounded are being sent out to South Africa to assist in connection with the troubles in Matabeliland.

Professor Behring is presenting the half of the Albert Levi prize, awarded to him, for the furtherance of research in connection with serum treatment in Germany.

The Russian Red Cross expedition to Abyssinia will consist of about six medical men, twenty-four nurses, and fifty assistants. Eight ambulances have already gone to the seat of war, and a portable hospital is to follow.

Through the death of Baron Hirsch, the metropolitan hospitals have lost a most munificent benefactor. He for some years devoted his winnings on the turf to them, and thus distributed over £70,000. It speaks well for the method upon which our institutions are conducted that Baron Hirsch gave so largely, for he had very definite views upon the subject of the administration of charities of all descriptions, and had studied the subject with care.

At the festival dinner in aid of the French Hospital, held last week, a collection of £2,500 was announced. M. Leon Geoffray, the French Chargé d'Affaires, presided, and amongst those present were the Lord Mayor, several members of foreign diplomatic circles, Sir William MacCormac, Dr. L. Vintras, and Mr. E. Rüffer, the secretary. The chairman alluded to the sympathies the Queen invariably showed to the French nation, and the Lord Mayor remarked that had the French Hospital not been established the English would have done their best to alleviate the sufferings of those belonging to the French nation.

The complaints against the spitting nuisance in public conveyances continue rife amongst us, yet nothing is done. In England we depend on the slow-working machinery of public opinion to alter such matters, and glory in our freedom from over-legislation. There are two sides to the advantages of our system. Elsewhere the State, coming to the aid of public opinion, is aiming a blow at the unhealthy and disgusting habit. The Health Department of New York City is causing passenger cars to be posted with placards forbidding the nuisance, and in St. Louis measures of a further-reaching nature are being adopted, and, to facilitate matters, spittoons are to be largely provided in public places. There is yet another point in which America is setting us a good example. It is universally recognised that our shops should be provided with seats for the attendants, yet nothing effectual in the matter has been done, publicly or privately. The Legislature of New Hampshire is now compelling all employers of females to provide seats for them. It only needs a certain amount of well-directed energy to do the same in England.

